# Cryptotanshinone Attenuates Oxygen-Glucose Deprivation/ Recovery-Induced Injury in an *in vitro* Model of Neurovascular Unit

**DOI:** 10.3389/fneur.2019.00381

**Published:** 2019-04-18

**Authors:** Hongye Zhao, Tiezheng Zheng, Xiaohan Yang, Ming Fan, Lingling Zhu, Shuhong Liu, Liying Wu, Changkai Sun

**Affiliations:** ^1^Department of Physiology and Key Laboratory of Brain Diseases of Liaoning Province, School of Basic Medical Sciences, Dalian Medical University, Dalian, China; ^2^Department of Physiology, School of Basic Medical Sciences, Qiqihar Medical University, Qiqihar, China; ^3^Department of Brain Protection and Plasticity, Institute of Basic Medical Sciences, Academy of Military Medical Sciences, Beijing, China; ^4^Department of Biomedical Engineering, Faculty of Electronic Information and Electrical Engineering & Research Center for the Control Engineering of Translational Precision Medicine, Dalian University of Technology, Dalian, China

**Keywords:** cryptotanshinone, oxygen-glucose deprivation/recovery, cerebral protection, neurovascular unit, apoptosis, BBB disruption

## Abstract

Cryptotanshinone (CTs), an active component isolated from the root of Salvia miltiorrhiza (SM), has been shown to exert potent neuroprotective property. We here established an oxygen-glucose deprivation/recovery (OGD/R)-injured Neurovascular Unit (NVU) model *in vitro* to observe the neuroprotective effects of CTs on cerebral ischemia/reperfusion injury (CIRI), and explore the underlying mechanisms. CTs was observed to significantly inhibit the OGD/R-induced neuronal apoptosis, and decease the activation of Caspase-3 and the degradation of poly-ADP-ribose polymerase (PARP), as well as the increase of Bax/Bcl-2 ratio in neurons under OGD/R condition. The inhibitory effects of CTs on neuron apoptosis were associated with the blocking of mitogen-activated protein kinase (MAPK) signaling pathway. CTs also remarkably ameliorated OGD/R-induced reduction of transepithelial electrical resistance (TEER) values and the increase of transendothelial permeability coefficient (Pe) of sodium fluorescein (SF) by upregulating the expression of ZO-1, Claudin-5, and Occludin in brain microvascular endothelial cells (BMECs), which might be related to the down-regulation of matrix metalloproteinase (MMP)-9 expression. Based on these findings, CTs may play a neuroprotective role in OGD/R injure in NVU models *in vitro* by inhibiting cell apoptosis and alleviating the damage of blood-brain barrier (BBB).

## Introduction

Stroke is the second leading cause of morbidity and mortality worldwide, with acute ischemic stroke (AIS) making up more than 80% of all the cases (1). AIS is caused by sudden interruption of artery supplying blood to the brain, usually resulting in a disorder of nervous system. Rapid restoration of blood supply is a critical therapeutic strategy for AIS, but it can bring secondary damage and further lead to more serious disturbance in the function of nervous system, called cerebral ischemia/reperfusion injury (CIRI). CIRI initiates a complex cascade of events, such as intracellular calcium overload, glutamate exitotoxicity, free radicals accumulation, excessive release of inflammatory mediators, DNA damage, blood-brain barrier (BBB) disruption, and apoptosis (2). Therefore, not only is CIRI involved in neurons but also in microvessels and gliacytes (3).

Hunting for an effective therapy for CIRI constitutes a challenging task in neuroscience for decades. Neuroprotection remains the central focus of CIRI treatment. Many mechanisms are involved in the origination and development of CIRI. Hence, neuroprotection targeting to a single therapeutic target is invalid. Based on this, LO EH and his colleagues proposed Neurovascular Unit (NVU) in 2003 (4). NVU is regarded as the basic structural and functional unit of brain, and mainly including neurons, astrocytes, microglia, microvascular endothelial cells, pericytes, even with basement membrane, and extracellular matrix. This concept not only shows the interactions among these types of cells, but also reflects their roles in the origination and development of brain diseases (5). So, NVU has become an important model for studying multi-target and multi-level therapy for brain diseases.

We here established an *in vitro* model of NVU, as described in previous reports (6). It is a co-culture system made up of three kinds of rat primary cells including brain microvascular endothelial cells (BMECs), astrocytes and neurons. This model can be used in brain research, potential drug targets screening and therapeutic drug discovery.

Salvia miltiorrhiza (SM) root has been commonly used to treat cardiovascular and cerebrovascular diseases in China and other Asian countries (7, 8). Cryptotanshinone (CTs), one of the major tanshinones isolated from the root of SM, is a kind of lipophilic compound and can pass through BBB (9, 10). CTs has various biological activities, such as anti-oxidation, anti-inflammation, anti-tumor, anti-apoptosis, anti-platelet aggregation activities, and so on (11–14). The previous studies demonstrated CTs possessed protective effects on the ischemic damage of multiple organs (15) and has the potential protective effects for CIRI (16). However, the protective effects of CTs on CIRI have not yet been confirmed, and its exact mechanism is unknown.

Oxygen-glucose deprivation/recovery (OGD/R) model is the most widely used in studies of CIRI *in vitro* (17), and many typical pathologic changes in CIRI were observed on OGD/R-injured NVU model (3). In the present study, we successfully established an OGD/R-injured NVU model *in vitro* to elucidate the potential protective effects of CTs on CIRI and explore its underlying mechanisms.

## Materials and Methods

### Animals

Sprague-Dawley (SD) rats were obtained from the Experimental Animal Center and housed in the Experimental Animal Center, Academy of Military Medical Sciences (Beijing, China). Newborn rats were sacrificed for isolating the primary cerebral astrocytes and neurons, and 120–150 g male rats were sacrificed for isolating the primary BMECs. All experiments followed an institutionally approved protocol in accordance with the China's Guidelines for Care and Use of Laboratory Animals.

### Preparation of CTs

CTs (*Mw*: 296.35, purity ≥98%) was purchased from the National Institute for Food and Drug Control (Beijing, China). CTs was dissolved in dimethyl sulfoxide (DMSO, Sigma-Aldrich, USA) to prepare for the stock solution with a concentration of 100 mM. The final concentration of DMSO in the testing solution was <0.1% (v/v) to prevent possible cytotoxicity.

### Reagents

MCDB 131 medium, DMEM, neurobasal A medium, microvascular growth supplement (MVGS) and B-27 were purchased from GIBCO (Thermo Fisher Scientific Inc., USA). DNase I, collagen type I and sodium fluorescein (*Mw*: 376.27, SF) were purchased Sigma (Sigma-Aldrich Co. LLC., USA). CCK-8 was purchased from Dojindo (Dojindo Laboratories, Japan). *In situ* cell death detection Kit (Fluorescein) and collagenase/dispase were purchased from Roche (Roche Applied Science, Germany). Clarity Western ECL Substrate kit were purchased from Bio-Rad (Bio-Rad Laboratories, Inc., USA). All the antibodies used in this research were purchased from CST (Cell Signaling Technology, Inc., USA), except the antibodies specific for ZO-1, Claudin-5, Occludin, MMP-2 and MMP-9 were from Abcam [Abcam (Shanghai) trading Co., Ltd., China].

### Isolation and Purification of Three Types of Rat Cerebral Cells

Primary rat BMECs were obtained from the cerebral cortex of 120–150 g rats according to previous reports (6) with some improvements. Primary rat cerebral astrocytes and neurons were obtained from cerebral cortex of 24 h newborn rats as previous reports (18) (Appendix 1).

The purified BMECs, astrocytes and neurons were used to establish the *in vitro* NVU model (Appendix 2).

### Establishment of OGD/R-Injured NVU Model *in vitro*

At first, we established a NVU model *in vitro* by referring to previous reports (6, 19) with a slight modification (Appendix 3).

The OGD/R treatment on NVU models *in vitro* were performed as previous descriptions (6). Briefly, the prepared NVU models *in vitro* were subsequently transferred into an anaerobic incubator (Coy laboratory, USA) with condition of 95% N_2_ and 5% CO_2_ at 37°C. Within the anaerobic incubator, the cell culture mediums were replaced with oxygen/glucose-free balanced DMEM without serum, which were previously saturated with 95% N_2_ and 5% CO_2_ at 37°C for 3 h. After OGD treatment for 2 h, the NVU models *in vitro* were switched to the normoxic incubator with high-glucose DMEM without serum for 24 h.

### Experimental Groups and Treatment

NVU models *in vitro* were randomly divided into 4 groups of Control, OGD2h/R24h and two doses of CTs (2.5 and 5.0 μM). Except for the Control group, each group was exposed to OGD2h/R24h. Drug treatment groups were treated with CTs (2.5 and 5.0 μM) for 3 h before OGD and during the OGD period (CTs was added into the culture mediums of insert). The experimental condition was established by a preliminary study involving different concentrations of CTs (0.32–10.0 μM) at different (3 and 24 h) pre- and post-hypoxia incubation periods (Appendix 4).

### Detection of Cell Viability

The cell viability of neurons was evaluated by cell counting kit-8 (CCK-8). The values were expressed as the percentage of living cells.

The cell viabilities of BMECs and astrocytes were tested by trypan blue stain after digestion with 0.25% trypsin-EDTA. The values were expressed as cells/cm^2^.

### TUNEL Assay

TUNEL assay was performed to analyze neuron apoptosis according to the manufacturer's instructions using *In situ* Cell Death Detection Kit. Finally, images were captured on a fluorescence microscope at × 100 and × 400 magnification. The neurons with green fluorescence were described as apoptotic neurons. The number of apoptotic neurons per view was counted using microscopy at × 400 magnification.

### Permeability Measurement of BBB by TEER and SF

The integrity of the BBB was measured via the transepithelial electrical resistance (TEER) assay using a Millicell ERS-2 Voltohmmeter (Millipore, USA) according to the protocol provided by the manufacturer. The TEER value of cell-free well was regarded as background TEER value. The final TEER value = (sample TEER value–background TEER value) × the area of insert membrane (4.52 cm^2^). The values were expressed as Ω × cm^2^.

Transendothelial permeability coefficient (Pe) of SF was performed as previously described (20). Briefly, the culture mediums in the inserts were switched to 1 ml Ringer-Hepes buffer containing a final concentration of 10 μg/ml SF. The inserts were transferred after 5, 15, 30, and 60 min to a new well with 2.5 ml Ringer-Hepes buffer, respectively. Hundred microliter culture mediums under the insert membrane were taken out at each observing time. The absorbance was measured by fluorospectrophotometer (Fluoroskan Ascent FL, Thermo Fisher Scientific Inc., USA, excitation wavelength: 485 nm; emission wavelength: 535 nm). The absorbance of cell-free well was regarded as background absorbance. Pe was calculated as previously described (21).

### Western Blotting

BMECs and neurons from NVU models were collected and lysed by RIPA buffer containing protease inhibitor, respectively. Protein concentration was determined by the BCA protein assay kit. Equivalent amounts of proteins from each group were subjected to SDS-PAGE gel electrophoresis, and transferred onto polyvinylidene fluoride (PVDF) membranes (Millipore, USA). After being blocked with 5% non-fat milk in TBST buffer for 1 h, the PVDF membranes were incubated with primary antibody at 4°C overnight. The primary antibodies used in this study were rabbit anti-Caspase-3 (1:1,000), rabbit anti-PARP (1:1,000), rabbit anti-Bax (1:1,000), rabbit anti-Bcl-2 (1:1,000), rabbit anti-p-ERK1/2 (1:1,000), rabbit anti-ERK1/2 (1:1,000), rabbit anti-p-JNK (1:1,000), rabbit anti-JNK (1:1,000), rabbit anti-p-p38 (1:1,000), rabbit anti-p38 (1:1,000), rabbit anti-ZO-1 (1:1,000), rabbit anti-Occludin (1:1,000), mouse anti-Claudin-5(1:2,000), rabbit anti-MMP-2 (1:1,000), rabbit anti-MMP-9 (1:1,000), rabbit anti-β-actin (1:1,000). After three washes with TBST buffer, the membranes were incubated with goat anti-mouse HRP or goat anti-rabbit HRP-conjugated IgG secondary antibodies (1:3,000) for 1 h each at room temperature. Protein bands were visualized with an Clarity Western ECL Substrate kit. The density of the bands was quantified using Image J software (National Institutes of Health, USA).

### Statistical Analysis

Data were expressed as the mean ± SD from at least three independent experiments. Data were analyzed by one-way analysis of variance (ANOVA) followed by Tukey's *post hoc* test using GraphPad Prism 6.0 software (GraphPad, La Jolla, CA, USA). Significant difference was accepted at *P* < 0.05.

## Results

### The Effects of CTs on Cell Viability and Cellular Apoptosis in Neurons in OGD/R-Injured NVU Model *in vitro*

**Figure 1:**
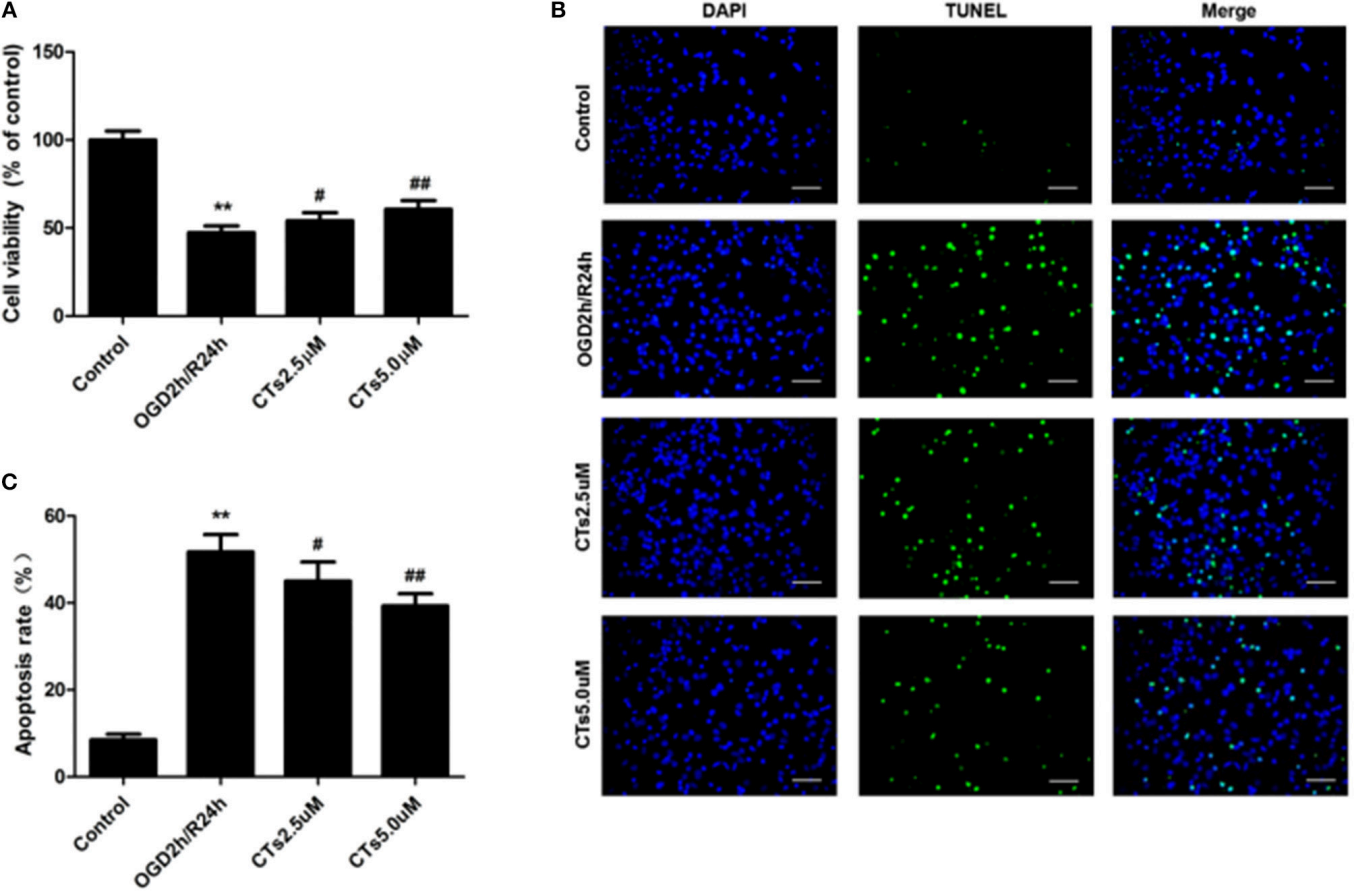
The effects of CTs on cell viability and cellular apoptosis in neurons in OGD/R-injured NVU model *in vitro*. **(A)** Cell viability was measured using the CCK-8 assay (*n* = 4). The cell viability of neurons was calculated by dividing the optical density of samples with the optical density of control. **(B)** Representative photomicrographs of apoptotic neurons were determined by TUNEL staining. Green fluorescence shows TUNEL-positive nuclei; blue fluorescence shows nuclei of total neurons. Scale bars: 25 μm. **(C)** The neuronal apoptosis rate was presented as the percentage of TUNEL-positive neurons. The percentage of TUNEL-positive neurons was calculated by dividing the number of TUNEL positive apoptotic neurons with the total number of neurons in 5 high-power fields. Data are presented as the mean ± SD. ^**^P < 0.01 vs. Control group; ^#^*P* < 0.05, ^##^*P* < 0.01 vs. OGD2h/R24h group. CTs, cryptotanshinone.

Compared with the neurons in control group, cell viability was significantly reduced to 47.35 ± 3.80% after OGD/R treatment (*P* < 0.01). CTs pre-treatment was able to increase the cell viability in neurons. The cell viability in CTs 2.5 and 5.0 μM group neurons were respectively, 54.10 ± 4.54% and 60.56 ± 5.01% (*P* < 0.05 and *P* < 0.01, Figure 1A). Meanwhile, the neuronal apoptosis was detected by TUNEL assay after OGD/R. In Control group, only about 8.50 ± 1.30% neurons were of apoptotic phenotype. This phenotype of neurons was remarkably increased to 51.73 ± 3.99% in the OGD2h/R24h group (*P* < 0.01). Pre-treatment with CTs 2.5 and 5.0 μM could protect against OGD/R induced apoptosis. The apoptotic neurons were significantly decreased to 45.00 ± 4.44% and 39.33 ± 2.78% respectively (*P* < 0.05 and *P* < 0.01, Figures 1B,C).

### The Effects of CTs on Apoptosis-Related Proteins Expressions in Neurons in OGD/R-Injured NVU Model *in vitro*

**Figure 2:**
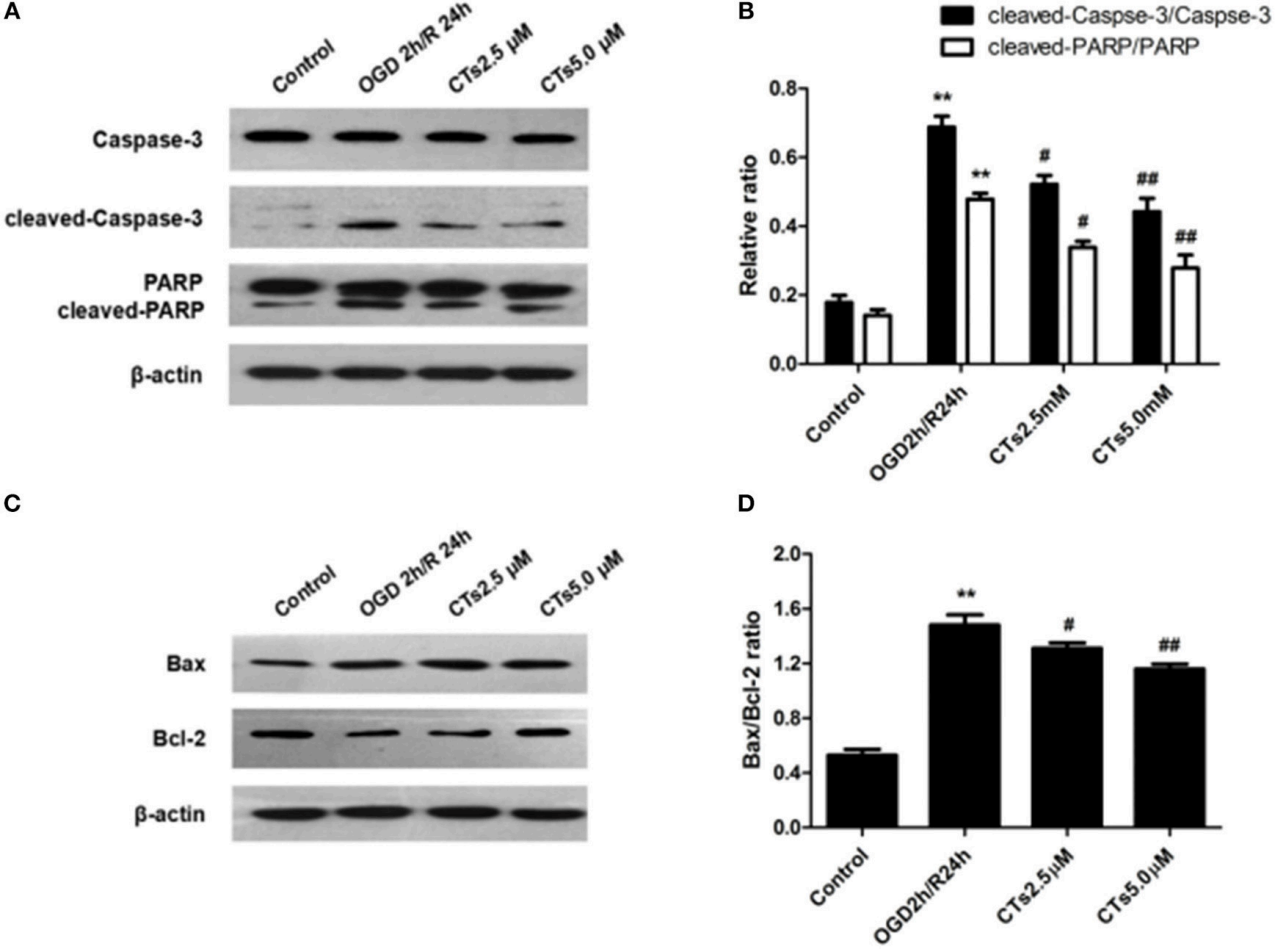
The effects of CTs on apoptosis-related proteins expressions in neurons in OGD/R-injured NVU model *in vitro*. **(A)** Representative Western blots showing the levels of Caspase-3, cleaved-Caspase-3(17, 19 kD), PARP and cleaved-PARP in neurons. **(B)** Relative density refers to the ratio of cleaved-Caspase-3(17, 19 kD) to Caspase-3 and cleaved-PARP to PARP (*n* = 4). **(C)** Representative Western blots showing the levels of Bax and Bcl-2 in neurons. **(D)** Relative density refers to the ratio of Bax to Bcl-2 (*n* = 4). The semiquantitative analyse results indicated that CTs protected neurons from apoptosis induced by OGD/R. Data are presented as the mean±SD. ^**^P < 0.01 vs. Control group; ^#^*P* < 0.05, ^##^*P* < 0.01 vs. OGD2h/R24h group. PARP, poly-ADP-ribose polymerase; Bcl-2, B-cell lymphoma 2; Bax, Bcl-2-associated X protein; CTs, cryptotanshinone.

The effect of CTs pre-treatment on apoptosis-related proteins expressions were confirmed by western blotting. As shown in Figure 2, it is clear that the Caspase-3 activation, PARP degradation and Bax/Bcl-2 ratio were significantly increased in neurons after OGD/R damage (all *P* < 0.01). However, the increased Caspase-3 activation, PARP degradation and Bax/Bcl-2 ratio were reduced by pre-treatment with CTs 2.5 and 5.0 μM (all *P* < 0.05 and *P* < 0.01).

### CTs Regulated the MAPK Signaling Pathways in Neurons in OGD/R-Injured NVU Model *in vitro*

**Figure 3:**
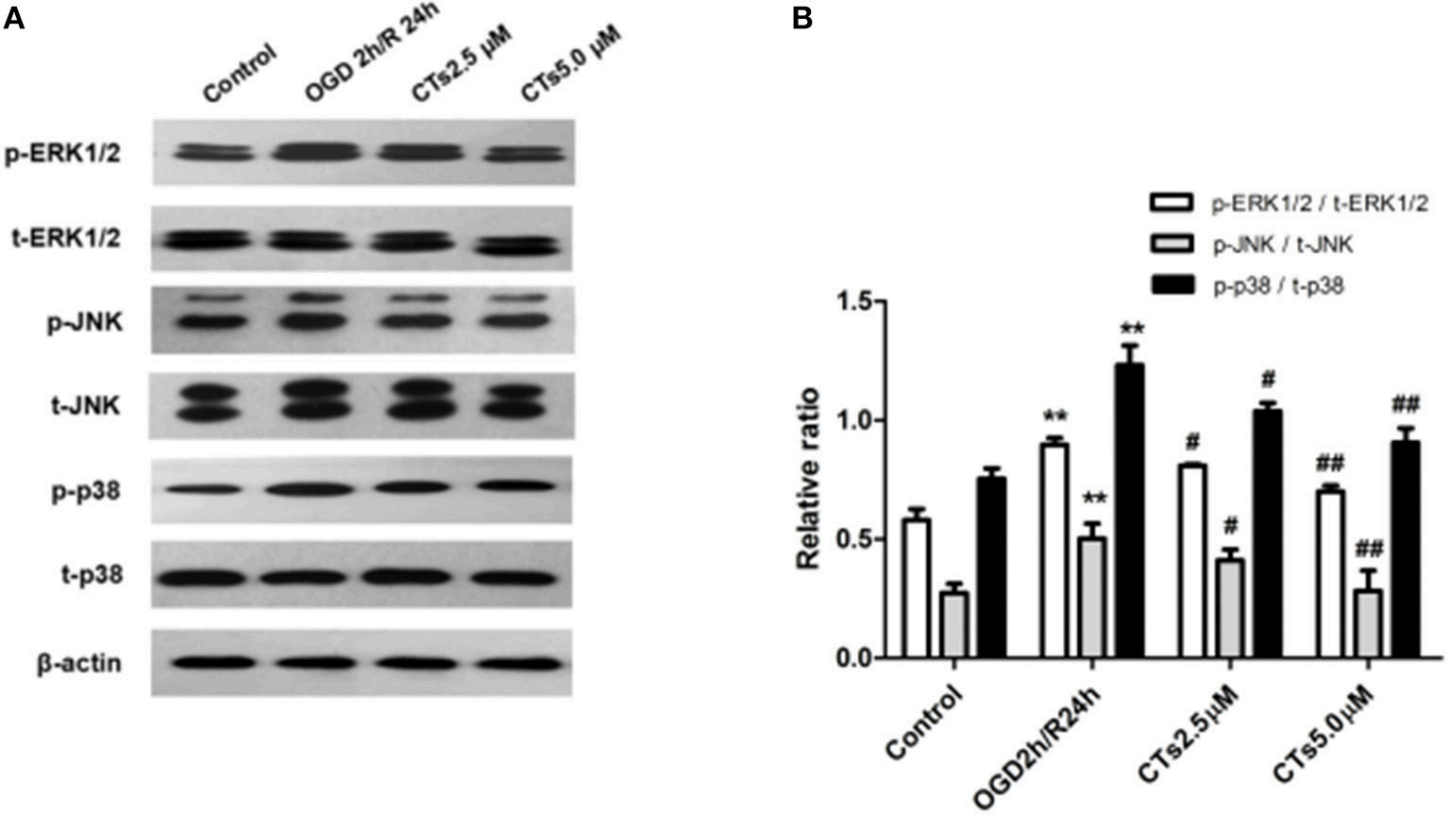
The effects of CTs on the MAPK signaling pathways in neurons in OGD/R-injured NVU model. **(A)** Representative Western blots showing the levels of p-ERK1/2, p-JNK, p-p38, t-ERK1/2, t-JNK, and t-p38 in neurons. **(B)** Relative density refers to the ratio of p-ERK1/2 to t-ERK1/2, p-JNK to t-JNK and p-p38 to t-p38 (*n* = 4). The semiquantitative analyses suggested that CTs pre-treatment obviously reduced the phosphorylation of ERK1/2, JNK and p38 in OGD/R injury neurons, thus regulated cell apoptosis. Data are presented as the mean ± SD. ^**^P < 0.01 vs. Control group; ^#^*P* < 0.05, ^##^*P* < 0.01 vs. OGD2h/R24h group. ERK1/2, extracellular regulated protein kinases1/2; JNK, c-Jun N-terminal kinase; p38, p38 mitogen-activated protein kinases; CTs, cryptotanshinone.

To assess whether CT pre-treatment would modulate the mitogen-activated protein kinase (MAPK) signaling pathways *in vitro*, we also examined the expression of total proteins [total extracellular regulated protein kinases1/2 (t-ERK1/2), total c-Jun N-terminal kinase (t-JNK), and total p38 mitogen-activated protein kinases (t-p38 MAPK)] and their phosphorylation (p-ERK1/2, p-JNK1/2, and p-p38 MAPK) on this pathway by western blotting. The results showed that there was a significant upregulation of p-ERK1/2, p-JNK, and p-p38 MAPK in neurons after OGD/R treatment (all *P* < 0.01). Pre-treatment with CTs 2.5 and 5.0 μM significantly downregulated p-ERK1/2, p-JNK, and p-p38 MAPK (all *P* < 0.05 and *P* < 0.01, Figures 3A,B).

### CTs Improved the BBB Function in OGD/R-Injured NVU Model *in vitro*

**Figure 4:**
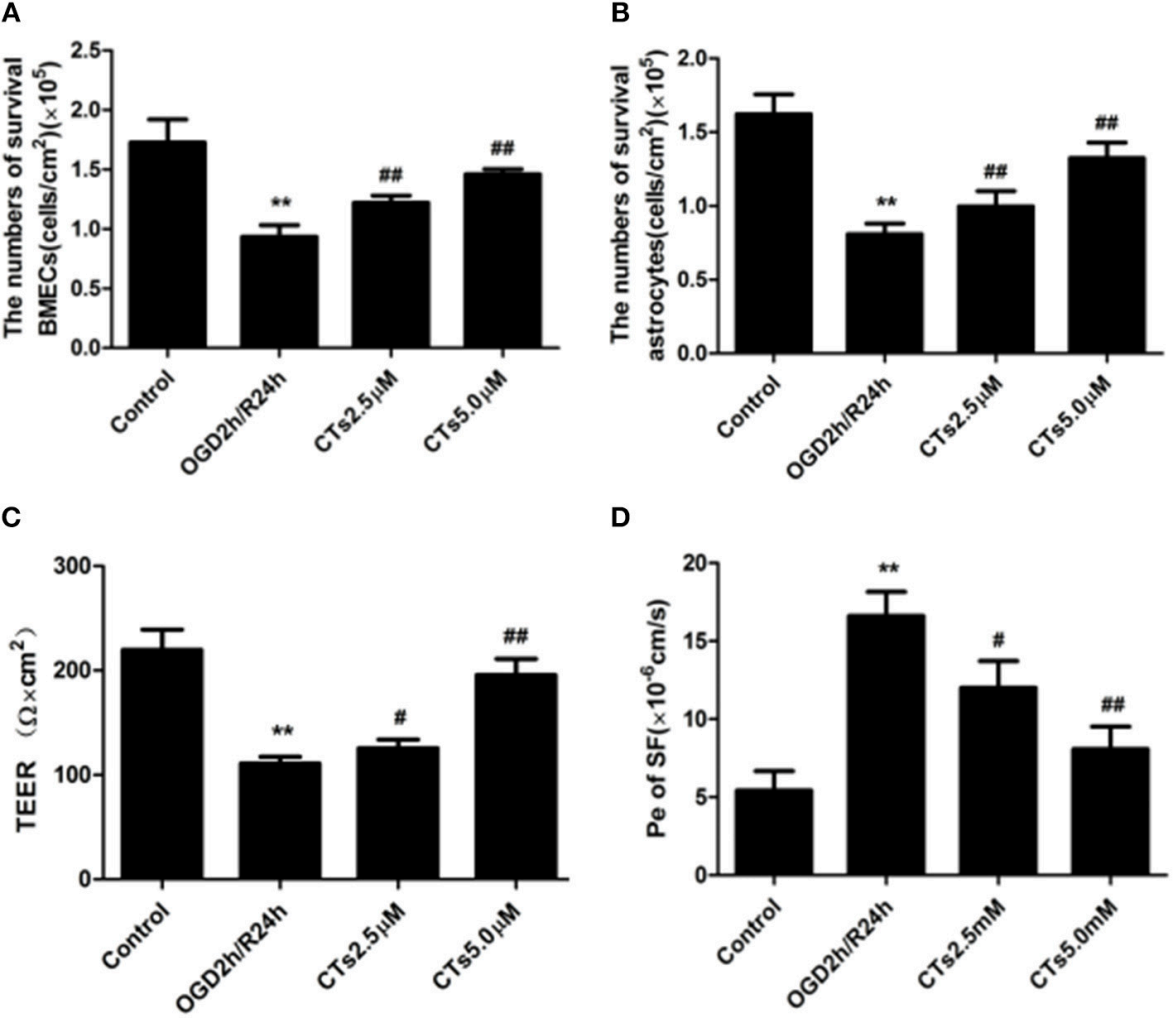
CTs improved the BBB function in OGD/R-injured NVU model. **(A,B)** The numbers of survival BMECs and astrocytes were detected by trypan blue stain.The values were expressed as cells/cm^2^ (*n* = 4). **(C)** TEER value was measured using a Millicell ERS-2 Voltohmmeter. The values were calculated according to the corresponding formula, and were expressed as Ω × cm^2^ (*n* = 4). **(D)** Pe of SF was calculated according to the corresponding methods. Pe were expressed as × 10^−6^cm/s (*n* = 4). The detection results showed CTs had an effect of protecting BBB function from OGD/R injury. Data are presented as the mean ± SD. ^**^P < 0.01 vs. Control group; ^#^*P* < 0.05, ^##^*P* < 0.01 vs. OGD2h/R24h group. BMECs, brain microvascular endothelial cells; TEER, transepithelial electrical resistance; Pe, permeability coefficient; SF, sodium fluorescein; CTs, cryptotanshinone.

BMECs and astrocytes are major cells that that comprise the blood-brain barrier. The TEER values and the Pe of SF can reflect paracellular permeability of BMECs. After OGD/R treatment, compared with Control group, the numbers of survival BMECs and astrocytes (both *P* < 0.01, Figures 4A,B) and the TEER value (*P* < 0.01, Figure 4C) significantly decreased, while Pe of SF (*P* < 0.01, Figure 4D) increased significantly, demonstrating the BBB integrity was destroyed. The above changes could be markedly reversed by pre-treatmet with CTs 2.5 and 5.0 μM (all *P* < 0.05 and *P* < 0.01).

### The Effects of CTs on Tight Junction Proteins and Matrix Metalloproteinases in BMECs in OGD/R-Injured NVU Model *in vitro*

**Figure 5:**
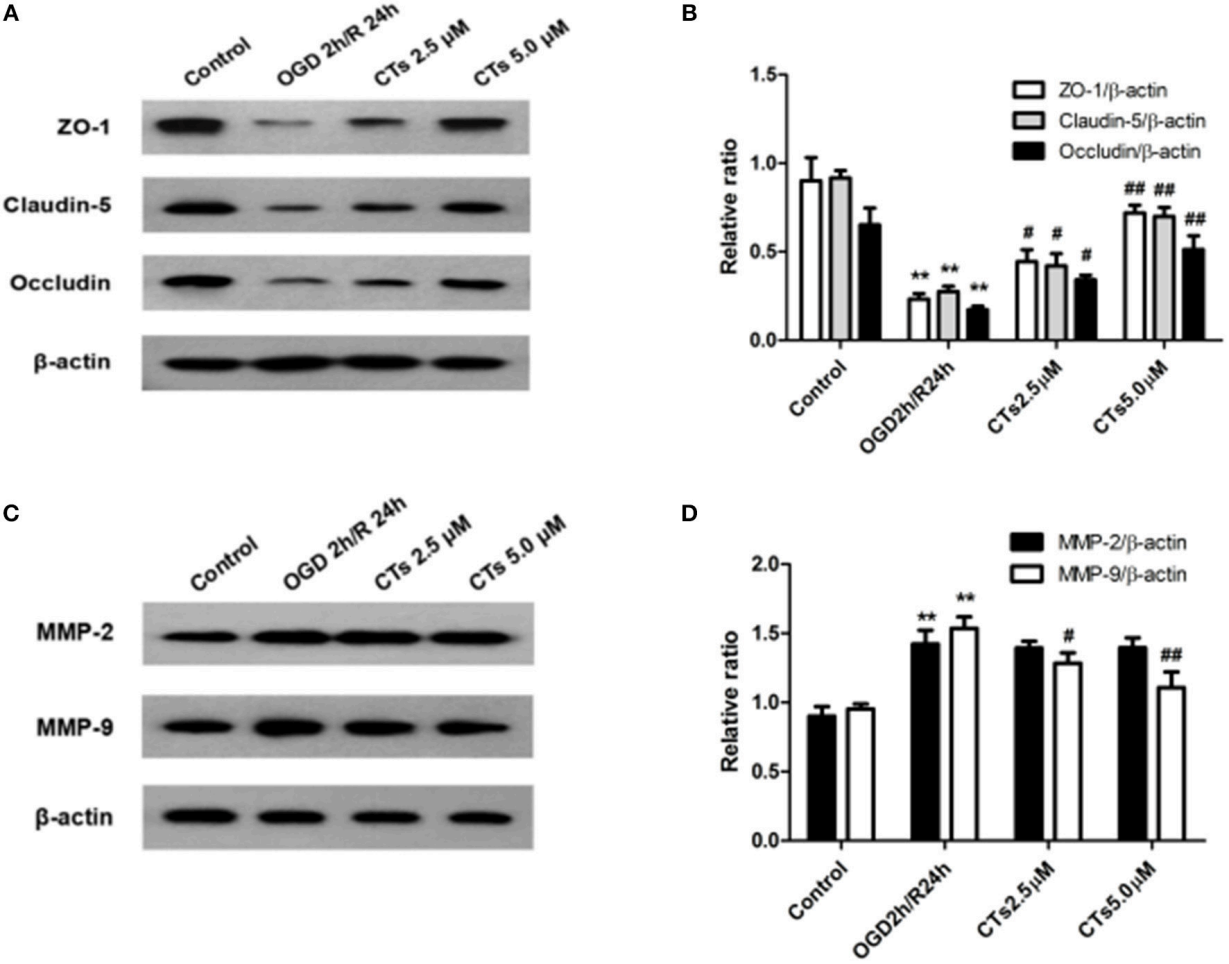
The effects of CTs on tight junction proteins and matrix metalloproteinase in BMECs in OGD/R-injured NVU model. **(A)** Representative Western blots showing the levels of ZO-1, Claudin-5, and Occludin in BMECs. **(B)** Relative density refers to the ratio of ZO-1, Claudin-5, and Occludin to β-actin. **(C)** Representative Western blots showing the levels of MMP-2 and MMP-9 in BMECs. **(D)** Relative density refers to the ratio of MMP-2 and MMP-9 to β-actin (*n* = 4). The semiquantitative analyses suggested CTs upregulated the expression of tight junction proteins by downregulating the expression of MMP-9 in OGD/R injury BMECs. Data are presented as the mean ± SD. ^**^P < 0.01 vs. Control group; ^#^*P* < 0.05, ^##^*P* < 0.01 vs. OGD2h/R24h group. BMECs, brain microvascular endothelial cells; ZO-1, Zonula occludens-1; MMP-2, matrix metalloproteinase-2; MMP-9, matrix metalloproteinase-9; CTs, cryptotanshinone.

To determine the effects of CTs pre-treatment on the tight junction proteins (TJPs) between endothelial cells, the levels of tight junction proteins (TJPs), ZO-1, Claudin-5 and Occludin, in BMECs were determined by Western blotting. As shown in Figures 5A,B, the protein expression levels of ZO-1, Claudin-5 and Occludin were significantly decreased after OGD/R treatment (all *P* < 0.01). Pre-treatmet with CTs 2.5 and 5.0 μM up-regulated obviously all of the expression levels of those proteins (all *P* < 0.05 and *P* < 0.01). Moreover, to explore the potential mechanisms of CTs on the protection of BBB function, the expression of matrix metalloproteinase-2 and -9 (MMP-2 and -9) were also examined by western blotting in the BMECs. The western blotting results exhibited that the expression levels of MMP-2 and -9 obviously increased after OGD/R injury (both *P* < 0.01**)**. The expression levels of MMP-9 significantly decreased in CTs 2.5 and 5.0 μM group (*P* < 0.05 and *P* < 0.01, Figures 5C,D). The expression of MMP-2 decreased in CTs 2.5 and 5.0 μM group although the difference was not significant (*P* >0.05).

## Discussion

AIS is induced by temporary occlusion of cerebral artery supplying blood. Reperfusion remains the critical therapeutic strategy for limiting brain injury following AIS, but the rapid restoration of blood flow is frequently associated with a serious secondary brain injury, called CIRI (22). AIS, particularly CIRI, triggers multiple cell signaling pathways in the brain, which may lead to neuron survival or damage (23, 24). However, the mechanisms involving neuronal fate following CIRI are complex and not fully clear. There is increasing evidence to show that cell apoptosis and disruption of the BBB play vital roles in neuron damage after CIRI (25–27).

CTs, one of the main active components of SM root, has already been shown to exert potent neuroprotective and antiapoptotic properties (28, 29). CTs could attenuates CIRI through inhibiting thrombosis formation, platelet aggregation and activation of PLC/PKC signaling pathway (8). CTs protected primary cortical neurons from glutamate-induced neurotoxicity through the activation of PI3K/Akt signaling pathway (9). Recent evidence has further shown that CTs exhibits a protective effect against cerebral stroke through suppressing the PI3K/AKT-eNOS signaling pathway (16). Taken together, these studies clearly show that CTs protects against CIRI and suggest that modulation of anti-apoptotic signaling cascades might be one of the molecular mechanisms of its neuroprotective effect.

**Figure 6:**
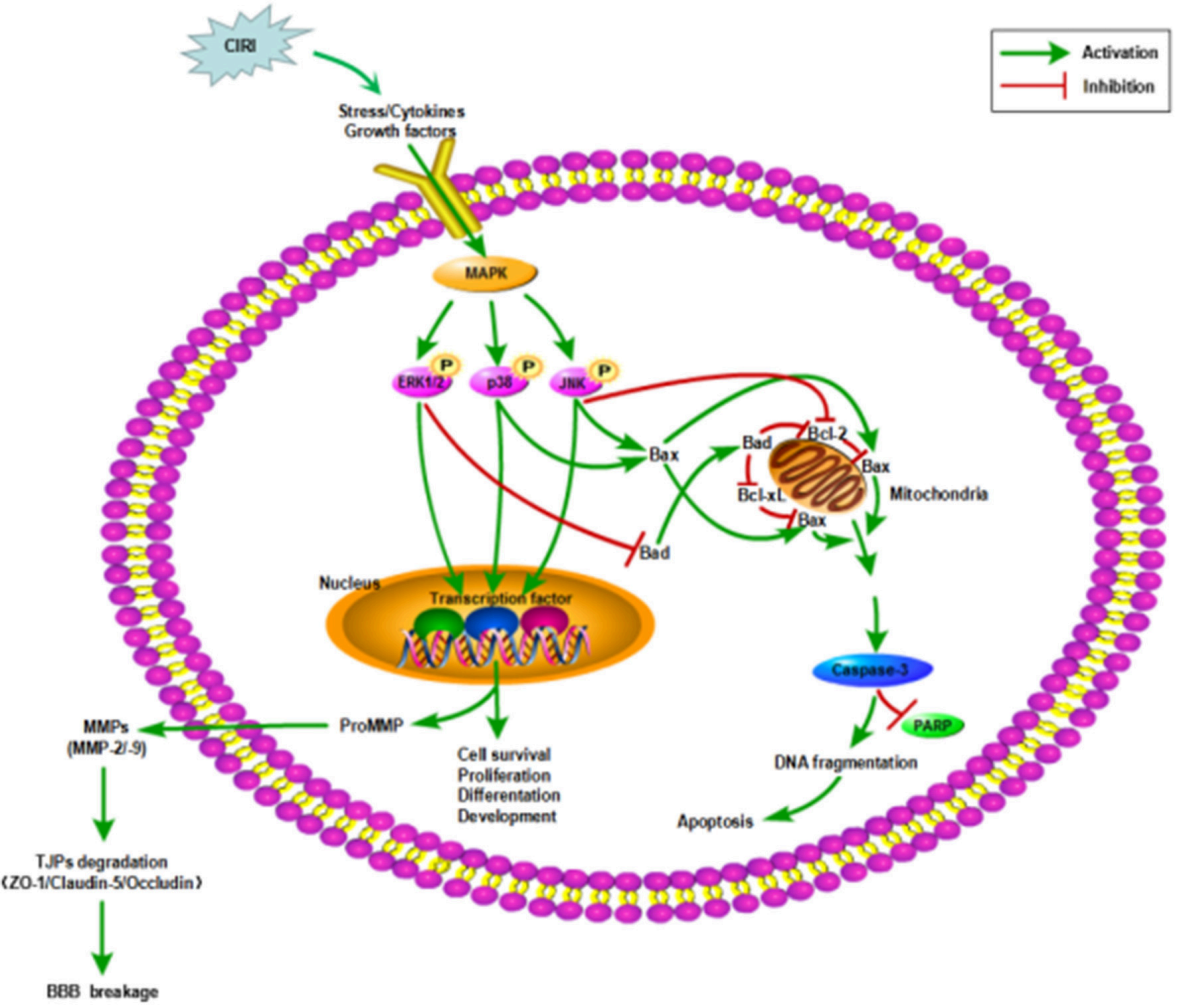
Mechanisms in CIRI inducing neuronal apoptosis and BBB disruption by activiating MAPK signaling pathways. AIS may lead to CIRI causing the release of proinflammatory cytokines and free radicals at the neurovascular unit activating MAPK signaling pathways. MAPK signaling pathways, including ERK1/2, JNK and p38 MAPK, participate in the regulation of neuronal survival or apoptosis and the activation of MMPs. Then MMPs activation disrupts the BBB integrity by degrading TJPs. ZO-1, Claudin-5 and Occludin are three of the important TJPs associated proteins. AIS, acute ischemic stroke; CIRI, cerebral ischemia/reperfusion injury; BBB, blood-brain barrier; MAPK, mitogen-activated protein kinase signaling pathways; ERK1/2, extracellular regulated protein kinases1/2; JNK, c-Jun N-terminal kinase; p38, p38 mitogen-activated protein kinases; Bcl-2, B-cell lymphoma 2; Bcl-xL, B-cell lymphoma-extra large; Bax, Bcl-2-associated X protein; Bad, Bcl-2-associated death protein; PARP, poly-ADP-ribose polymerase; TJPs, tight junctions proteins; ZO-1, Zonula occludens-1; MMP-2/-9, matrix metalloproteinase-2/-9.

In this study, we used OGD/R-injured NVU model *in vitro* to further investigate the neuroprotective effects of CTs on CIRI as well as the underlying mechanisms by focusing on MAPK signaling pathways, as these pathways were closely related to cell survival and apoptosis (Figure 6). In line with previous studies, our results indicated that CTs had significant neuroprotective effects against CIRI.

Cell apoptosis is a major characteristic of CIRI. Reducing neuronal apoptosis could minimize or even prevent the occurrence of CIRI (25). In this study, we found that CTs pre-treatment could decrease the apoptotic rate in the neurons of NVU model *in vitro* exposed to OGD/R. Meanwhile, we further investigated the Bax/Bcl-2 ratio. The Bcl-2 family member Bax was markedly up-regulated after OGD/R, which resulted in the release of cytochrome *c* to the cytosol. Bcl-2 exerts the anti-apoptosis efficiency through inhibiting the function of Bax (30, 31). We found that the OGD/R-induced up-regulation of Bax/Bcl-2 ratio could be reversed by CTs pre-treatment. Moreover, we analyzed the activation of Caspase-3 and the degradation of PARP. Cytochrome *c* release triggers the cleavage of Caspase-3 to activate the Caspase-3 protein, which results in DNA fragmentation and cell apoptosis by cleaving PARP (31, 32). Our data revealed that pre-treatment with CTs prevented the OGD/R-induced increase in cleaved-Caspase-3 and cleaved-PARP. Our findings suggested that CTs could protect neurons from apoptosis induced by OGD/R.

Neuronal damage after CIRI is usually caused by oxidative stress, inflammation response, or mitochondrial dysfunction, and activates ultimately an apoptotic cascade. MAPK signaling pathways, as both targets and mediators of CIRI, are involved in neuronal survival or apoptosis regulation after stroke (33–35). Thus, inhibiting or regulating the expression and activity of MAPK signalings may constitute novel therapeutic strategies for CIRI (36, 37). Recent studies have found that several drugs might play protective roles in CIRI by inhibition of MAPK signaling pathways (38, 39). The MAPK family includes extracellular signal-regulated kinase 1/2 (ERK1/2), c-Jun amino terminal kinase (JNK) and p38 MAPK. These pathways are activated through phosphorylation by upstream kinases recruited through diverse extracellular signaling events. Our results demonstrated that the phosphorylation of ERK1/2, JNK, and p38 MAPK was significantly increased in the neurons of NVU model *in vitro* exposed to OGD/R. Pre-treatment with CTs resulted in decreased phosphorylation of all the three molecules. These results suggested that MAPK signaling pathways could be involved in the neuroprotective effects of CTs. It is known that, the activated ERK1/2, JNK and p38 MAPK mainly mediate the cellular stress in CIRI by phosphorylating intracellular enzymes, transcription factors, and cytosolic proteins involved in cell survival and apoptosis (40–42). Our results indicated that the protective effects of CTs against neuronal apoptosis induced by OGD/R could associate with suppressing the activation of MAPK signalings.

In addition, the activation of MAPK signaling pathways is associated with BBB damage (Figure 6) and aggravates CIRI by promoting the production of inflammatory cytokines (43, 44). BBB disruption is a hallmark of stroke and a mediator of CIRI and stroke progression (45). Restoration of the BBB can relieve neuronal damage caused by CIRI (46). Our findings demonstated that CTs could suppress the activation of MAPK signalings in neurons. Therefore, we hypothesized that CTs might have a beneficial effect on CIRI-induced BBB dysfunction. As we know, BBB, is mainly made up of BMECs and astrocytes, plays a critical role in maintaining the microenvironment of brain (18). Accumulating evidence has indicated that BBB dysfunction is a key event during the progression of CIRI (47). Therefore, protecting BBB from disruption may be a promising strategy for prevention and treatment of CIRI (48). In the current study, we found that the cell viabilities of BMECs and astrocytes of NVU model *in vitro* were significantly reduced after OGD/R. Pre-treatment with CTs increased notably the cell viabilities of BMECs and astrocytes. Moreover, the permeability measurement of BBB by TEER and SF showed that CTs pre-treatment remarkably reversed the leakage of BBB in the OGD/R-injured NVU model *in vitro*. These results suggested that CTs could effectively improve BBB integrity during OGD/R.

Tight junctions proteins(TJPs) between BMECs participate in forming the BBB and are composed of Zonula Occludens (ZO), Claudins and Occludin, all of which play important roles in regulating BBB permeability and function (49, 50). And there is growing evidence that the disruption of ZO-1, Claudin-5, and Occludin led to the functional changes of TJs (51–53). In this study, we found that the expression of ZO-1, Claudin-5 and Occludin was significantly down-regulated in BMECs of NVU model *in vitro* after OGD/R. Pre-treatment with CTs could maintain the expression of these three kinds of TJPs. These data confirmed that CTs could attenuate the BBB damage during CIRI by maintaining the TJPs expression in BMECs. Furthermore, alterations of MMPs also affect the function of the BBB (54). MMPs, a family of zinc- and calcium-dependent enzymes, disrupt the BBB integrity by degrading TJPs (55–57). The expression of MMPs is only low level in normal brain tissue, but many MMPs are activated and their levels increase after ischemic stroke (58). Members of the MMPs family, specifically MMP-2 and MMP-9 are involved in the breakdown of the BBB and increased levels of these MMPs have been observed during CIRI in stroke (59, 60). Consistent with these observations, we found the increase of MMP-2 and -9 protein levels in BMECs of NVU model *in vitro* exposed to OGD/R. CTs pre-treatment down-regulated the expression of MMP-9, but not MMP-2. These data further supported that CTs maintained the expression of ZO-1, Claudin-5 and Occludin, probably during OGD/R via inhibiting protein expression of MMP-9. MMPs expression is tightly regulated at the transcriptional and post-translationa levels. However, the exact mechanism is unclear. At present, it is believed that multiple signaling pathways are involved in this complex regulatory process, such as MAPK, NF-κB, and PI3K/Akt signaling pathway (61–63), which may be the reason why CTs only down-regulated the expression of MMP-9. Further study is required to explore the functional relationship between the regulation of MMP-2 expression and BBB integrity and health.

## Limitations

Several limitations should be acknowledged for the present study. Again further studies are needed to reveal the effects of CTs on oxidative stress, inflammation and the other two types of cells apoptosis in the NVU model during OGD/R injury, BMECs and astrocytes for instance.

## Conclusions

Despite the above limitations, we indicate that the protective mechanism of CTs against OGD/R damage might exert via inhibiting neuron apoptosis and attenuating BBB disruption. Furthermore, we also clarified that CTs inhibited neuronal apoptosis possibly by blocking the activation of MAPK signaling pathways, and CTs alleviating BBB disruption may associated with the regulation of TJPs and MMP-9 in our experiment. Accordingly, CTs will represent a novel and potent candidate for the treatment of CIRI in the future.

## Ethics Statement

This study was carried out in accordance with the recommendations of China's Guidelines for Care and Use of Laboratory Animals, Medical Ethics Committee of Dalian Medical University. The protocol was approved by the Medical Ethics Committee of Dalian Medical University.

## Author Contributions

CS, LZ, and MF were involved in designing the study, interpreting the data and writing the manuscript. HZ, TZ, and XY performed the majority of the experiments. SL and LW contributed to the analysis of the data.

### Conflict of Interest Statement

The authors declare that the research was conducted in the absence of any commercial or financial relationships that could be construed as a potential conflict of interest.
