# Supplementary material for: Cryptotanshinone Attenuates Oxygen-Glucose Deprivation/ Recovery-Induced Injury in an in vitro Model of Neurovascular Unit
Source: Front Neurol. 2019 Apr 18;10:381. doi: 10.3389/fneur.2019.00381 (PMC6482155; doi:10.3389/fneur.2019.00381)
Supplement: Supplementary file 1 [file Data_Sheet_1.docx]

**Appendices**

1. ***Isolation and purification of three types of rat cerebral cells***

Primary rat BMECs were obtained from the cerebral cortex of 120-150 g rats. Brifely, meninges, large vessels and white matter were removed carefully and grey matter was minced into small pieces of approximately 1.0 mm^3^ in ice-cold D-Hanks. After centrifuged at 380 (×g) for 5 min, the precipitation layer was added with D-Hanks, which contained collagenase/dispase (1.0 mg/ml) and DNase I (10.0 μg/ml), and digested at 37 °C for 1 h. Cold DMEM with 10% FBS was added to terminate the digestion and then it was centrifuged for 5 min. The precipitate was re-suspended in 20% BSA and centrifuged at 1420 (×g) for 15 min. The obtained microvessels were digested in collagenase/dispase (1.0 mg/ml) and DNase I (10.0 μg/ml) at 37°C for 2 h. After terminating the digestion and centrifuging at 380(×g) for 5 min, the precipitate was re-suspended in 1 ml cold D-Hanks. Then BMECs were purified from the new suspension by percoll gradient centrifugation. at 1420 (×g) for 20 min. And then, the BMECs suspension was washed and centrifuged twice with D-Hanks liquid, re-suspended in MCDB 131 medium containing 5% FBS, 5% MVGS, 1% Hepes, L-glutamine (0.3 mg/ml), 1% penicillin-streptomycin solution and puromycin dihydrochloride (4 μg/ml). After the density adjustment, the BMECs were seeded on 25 cm^2^ plastic dishes pre-coated with collagen type I (2 ml) and incubated at 37°C and 5% CO_2_. After 48 h, the culture medium was replaced with the ordinary medium, which is the same as the previous medium only without puromycin dihydrochloride. From then on, the culture medium was changed every 3 days. When the confluence reached 80% (about the 6th day), the BMECs were further purified with 0.25% trypsin.

Cerebral astrocytes and neurons were obtained from cerebral cortex of 24 h newborn rats. Briefly, cerebral cortex were disaggregated in 0.25% trypsin-EDTA at 37°C for 30 min. After centrifuged at 380(×g) for 5 min, the precipitate was re-suspended in the DMEM with 10% FBS, and passed through 200-mesh sieve to get cells suspension. Then the filtrate was centrifuged at 380 (×g) for 5 min and then re-suspended with DMEM containing 10% FBS, 1% Hepes, L-glutamine (0.3 mg/ml), 1% penicillin-streptomycin solution and planted on 25cm^2^ plastic dishes at 37°C and 5% CO_2_. Then the culture medium was changed every 3 days. When the confluence reached 80% (about the 4-5th day), the plastic dish was shaken at 250 rpm for 18 h at 37 °C to purify the cells. The purified astrocytes were passaged by a brief treatment with 0.25% trypsin and the second passage was used to establish the *in vitro* NVU model.

The cultures of primary neurons were the same as that of primary astrocytes mentioned previously, but the cells were seeded in 6-well plates pre-coated with poly-L-lysine (0.1 mg/ml) at 37°C and 5% CO_2_. After the adherent cells growth, medium was changed into neurobasal A medium containing 2% B-27, 1% Hepes, L-glutamine (0.3 mg/ml) and 1% penicillin-streptomycin solution. On the third day, medium was replaced with new medium supplemented with Ara-C (5.0mg/ml) to inhibit the non-neuronal cell growth for 24 h. Then, the half of culture medium was changed every 2 days.

1. ***Immunophenotyping controlled the qualities of the primary cells and purities used in NVU models in vitro***

The purity of these cells was identified by immunohistochemical staining. Cells markers of factor VIII, GFAP, and MAP2 were selected for BMECs, astrocytes and neurons, respectively. And the cell nucleus were stained with 4,6-diamidino-2-phenylindole (DAPI). Images were captured on a fluorescence microscope (Olympus Corporation, Japan) at ×200 magnification. The purity of cells was calculated by the following formula: the rate of cell purity = the number of cells with DAPI^+^ and marker^+^/the number of cells with DAPI^+^.

As shown in Figure 1, the BMECs were identified by factor VIII, and the purity was beyond 99%. The astrocytes were detected by GFAP, and the purity was beyond 96%. The neurons were determined by MAP2, and the purity was beyond 95%.

The purity of cell achieved to 95% was considered to be used in the study.


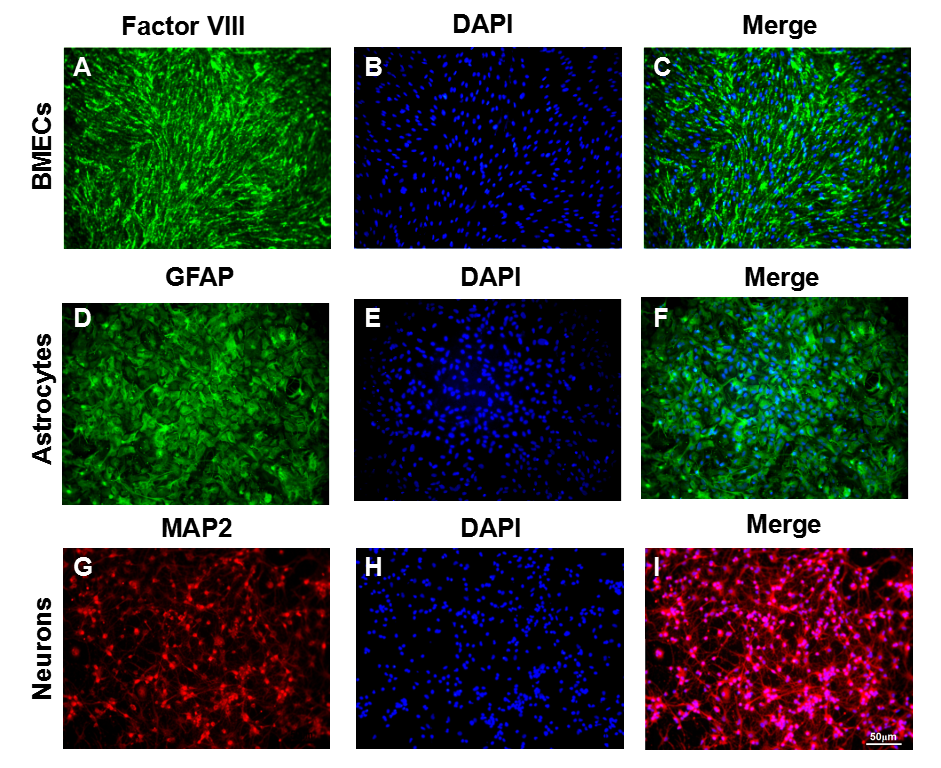


**Figure 1. Immunophenotyping controlled the qualities of the primary cells used in *in vitro* NVU models.** A-C. Immunophenotyping of BMECs were indentified by factor VIII. D-F. Immunophenotyping of astrocytes were indentified by GFAP. G-I. Immunophenotyping of neurons were indentified by MAP2. Scale bars: 50 μm.

1. ***Establishment of NVU model in vitro***

We established a NVU model *in vitro* with co-cultured rat BMECs, astrocytes and reurons using transwell insert (Millipore, USA). As shown in Figure 2, the purified astrocytes with 1.5×10^5^ cells/cm^2^ were seeded on the outside of insert membrane. After 2 h for astrocytes adhering, the insert was placed into an empty well. Then the BMECs with 1.0×10^5^ cells/cm^2^ were seeded in the inside of insert membrane. After 2-3 days, the insert was placed into the well where neurons had already grown with a seeding density of 0.5×10^5^ cells/cm^2^ for 2-3 days. After co-cultured for 3-5 days, the NVU model was established successfully.


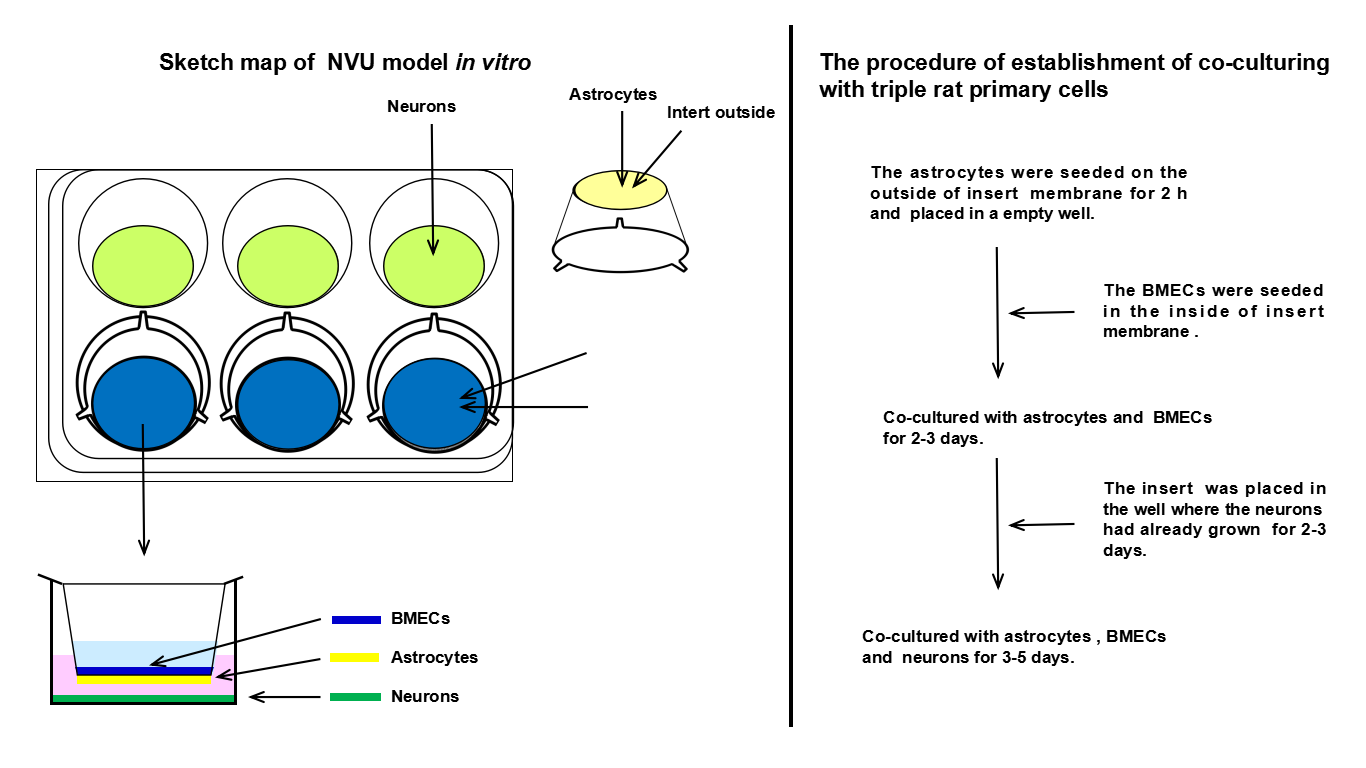


**Figure 2. The procedure of establishing NVU model *in vitro.***

1. ***Determination of treatment method and suitable dose***

Normally, lactate dehydrogenase (LDH) is abundant in cytoplasm, and can not pass through cell membrane. But if the cells are damaged or dead, LDH will be secreted out of the cell. Therefore, we detected the LDH activity in the culture fluid of NVU model as a cell OGD/R injury indicator.

We randomly divided the established NVU models *in vitro* into two groups of Pre-treatment and Post-treatment. Each treatment group included 8 groups of Control, OGD 2h/R 24h and six doses of CTs (0.32μM, 0.63μM, 1.25μM, 2.5μM, 5.0μM and 10.0 μM). Except for the Control group, each group was exposed to OGD2h/R24h. In the Pre-treatment groups, CTs groups were treated with CTs for 3 h before OGD and during the OGD period. In the Post-treatment groups, CTs groups were treated with CTs for 24 h after OGD (during the recovery period). CTs was added into the culture mediums of insert. After OGD2h/R24h, LDH activity was measured using a LDH assay kit (Jiancheng, Nanjing, China) according to the manufacturer’s instructions.

As shown in Figure 3, the LDH activity in the culture fluid of OGD2h/R24h Group significantly increased compared with those of Control group. Pre-treatment with CTs 2.5μM, 5.0μM and 10.0μM for 3 h significantly decreased the LDH activity in the culture fluid of NVU model. But there was not obviously difference between Post-treatment with CTs group and OGD2h/R24h group in the LDH activity in the culture fluid of the NVU model.

In conclusion, pre-treatment with CTs for 3h is the best treatment method to observe the effects of CTs on the OGD/R injury of NVU model *in vitro*. In the Pre-treatment groups, CTs 2.5 μM, 5.0 μM, and 12.5 μM were all the effective doses. In order to observe the intervention effects of CTs on OGD/R injury of NVU model *in vitro*, we selected 2.5 μM and 5.0 μM as the suitable doses of CTs, which could significantly reduce the OGD/R injure of NVU model with a good dose-effect relationship.

**
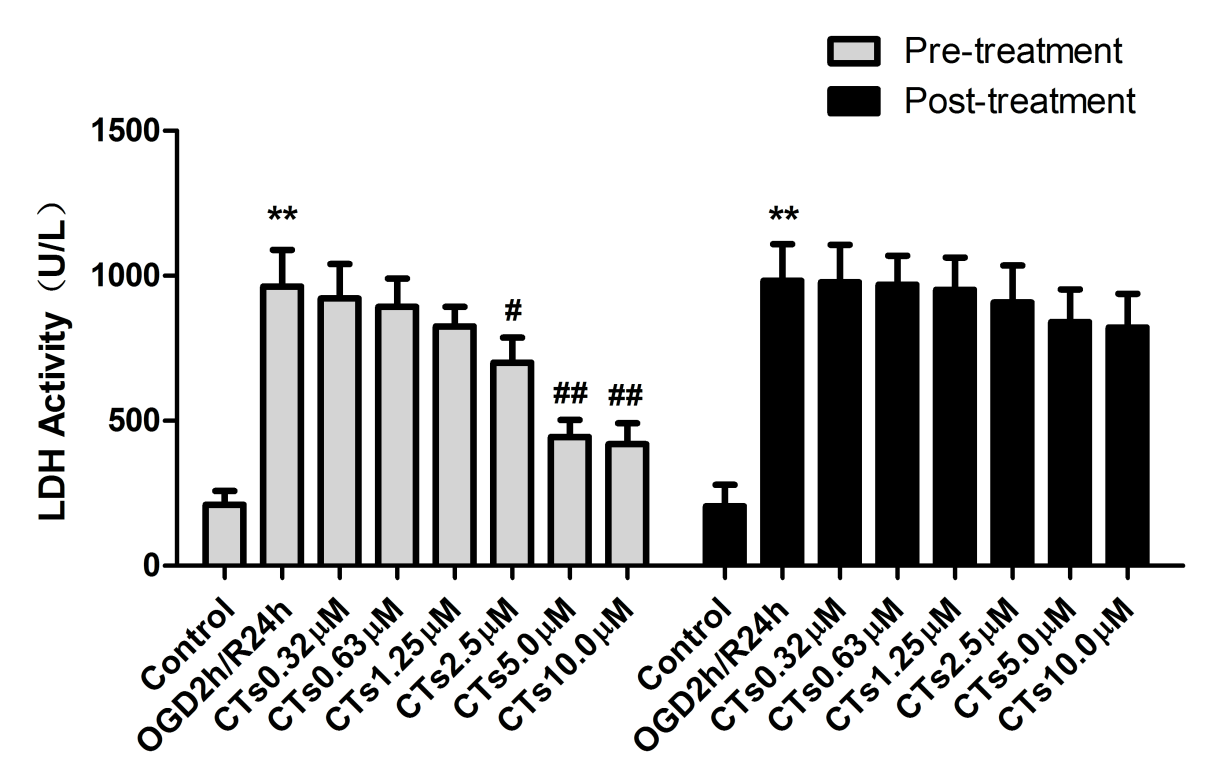
**

**Figure 3. The effects of CTs on LDH activity in the culture fluid of OGD/R-injured NVU model *in vitro*** LDH activity was measured using the LDH assay kit (*n*=4). Data are presented as the mean ± SD. ***P*<0.01 vs. Control group; ^#^*P*<0.05, ^##^*P*<0.01 vs. OGD2h/R24h group. LDH, lactate dehydrogenase; Pre-treatment, pre-treatment with CTs groups; Post-treatment, post-treatment with CTs groups; CTs, cryptotanshinone.
